# A Survey of Differential-Fed Microstrip Bandpass Filters: Recent Techniques and Challenges

**DOI:** 10.3390/s20082356

**Published:** 2020-04-21

**Authors:** Yasir I. A. Al-Yasir, Naser Ojaroudi Parchin, Ahmed M. Abdulkhaleq, Mustafa S. Bakr, Raed A. Abd-Alhameed

**Affiliations:** 1 Biomedical and Electronics Engineering, Faculty of Engineering and Informatics, University of Bradford, Bradford BD7 1DP, UK; N.OjaroudiParchin@bradford.ac.uk (N.O.P.); A.Abd@sarastech.co.uk (A.M.A.); R.A.A.Abd@bradford.ac.uk (R.A.A.-A.); 2 SARAS Technology Limited, Leeds LS12 4NQ, UK; 3 Institute of Microwave and Photonics, University of Leeds, Leeds LS2 9JT, UK; M.S.A.Bakr@leeds.ac.uk; 4 Department of Communication and Informatics Engineering, Basra University College of Science and Technology, Basra 61004, Iraq

**Keywords:** differential-fed filter, microstrip, RF, MW, 4G, 5G, bandpass

## Abstract

Differentially driven devices represent a highly promising research field for radio frequency (RF), microwave (MW), and millimeter-wave (mmWave) designers and engineers. Designs employing differential signals are essential elements in low-noise fourth-generation (4G) and fifth-generation (5G) communications. Apart from the conventional planar MW components, differential–fed balanced microstrip filters, as promising alternatives, have several advantages, including high common-mode rejection, low unwanted radiation levels, high noise immunity, and wideband harmonic suppression. In this paper, a comprehensive and in-depth review of the existing research on differential-fed microstrip filter designs are presented and discussed with a focus on recent advances in this research and the challenges facing the researchers. A comparison between different design techniques is presented and discussed in detail to provide the researchers with the advantages and disadvantages of each technique that could be of interest to a specific application. Challenges and future developments of balanced microstrip bandpass filters (BPFs) are also presented in this paper. Balanced filters surveyed include recent single-, dual-, tri-, and wide-band BPFs, which employ different design techniques and accomplish different performances for current and future wireless applications.

## 1. Introduction

In recent years, fourth-generation (4G) and fifth-generation (5G) wireless applications have been experiencing fast development [1,2,3,4,5,6,7,8,9,10,11,12,13,14,15,16]. Signal crosstalk, interference, and high costs have a big effect on the rapid development of radio frequency (RF) and microwave (MW) devices, while common-mode signal causes radiation power loss of up to 25% of the input power in the millimeter-wave (mmWave) spectrum (26–40 GHz) [17,18]. Over the last decade, many differential-fed devices, which provide high protection to interference signals, low RF noise, and a good degree of freedom, were increasingly in need of more attention and further studies [19]. Differential-fed filters [20,21], differential-fed power dividers [22,23], and differential-fed antennas [24,25,26] are the most widely used differentially driven microstrip devices. Therefore, in order to meet the increasing demands for multifunctional systems in the recent wireless applications such as 4G and 5G systems, differential-fed planar bandpass filters (BPFs) are highly required and recommended for these applications. However, many design techniques in many proposals and research articles have been accomplished on the differential-fed (balanced) microstrip BPFs over the last years [27,28,29,30,31,32,33,34,35]. With high data rate transmissions over channels, RF, MW, and mmWave systems operating with differentially driven ports represent a highly promising research topic for researchers, designers, and engineers. It is expected that more and more design techniques for balanced circuits will be proposed and developed in the next few years. It is worth mentioning that, compared to the single-band microstrip BPFs, traditional high-order multi-band microstrip BPFs present high insertion losses and less passband selectivity due to the metal resistance. Also, wide-band differential microstrip BPFs are essential in the new generations of wireless systems since they can provide higher data rate transmission and higher suppression for noise and interference signals compared with the narrow-band balanced microstrip BPFs. In our view, for passive differential-fed microstrip BPFs, the main important advantages can be summarized as follows:

- High noise immunity.
- High common-mode attenuation.
- High passband selectivity.
- Wide-stopband harmonic suppression.
- Low radiation power loss.
- Multi-function integration.
- High linearity.

So far, a few review papers have been introduced on the scop of balanced filters [36,37]. Arbelaez-Nieto et al. [36] reviewed and studied some basic concepts related to microwave balanced bandpass filter structures and discussed some alternative methods to design, simulate, and measure differentially fed microstrip BPFs. To simplify the design procedure for other researchers, the paper has presented the step by step developments of planar differentially driven BPFs. To fully describe differentially driven devices, mixed-mode reflection coefficients were presented and discussed generally and this can be applied whenever a designer has a multiport circuit. Since balanced filters have a pair of differential ports or a pair of two single-ended ports short-circuited to the ground, the authors have also proposed a design methodology for differential-fed microstrip filters using mixed-mode s-parameter conversion.

Feng et al. [37] proposed a survey on new balanced planar devices using dual-mode ring resonator structures. A comparison between recently proposed balanced RF components using different design techniques was also presented and discussed. The paper has shown that the common-mode attenuation can be up to five times the passbands for the differential-fed filters. Furthermore, using dual-mode ring resonators can enhance the filtering performance for differential-fed power dividers and crossovers, and this can minimize the system size and decrease the radiation power loss. The dual-mode ring resonator circuits can also offer more compact sizes by employing multilayer techniques [38], and this will lead to the development of a high suppression of the common-mode noise for high data rate transmission and high-frequency applications.

Unlike other presented reviews, our paper aims to survey and compare several design techniques of differential-fed microstrip BPFs by focusing on recent advances and challenges. Differential-fed microstrip BPFs can be classified according to their performance into four categories, as follows:

- Single-band differential microstrip BPFs.
- Dual-band differential microstrip BPFs.
- Tri- and quad-band differential microstrip BPFs.
- Wide-band and ultra-wideband differential microstrip BPFs.

Each category can be performed using different design techniques and structures according to the system requirements and limitations. All these aspects will be detailed in the following sections of this research. This survey is organized as follows: Section 2 reviews single-band differential BPFs. Section 3 reviews dual-band differential BPFs. Section 4 reviews tri- and quad-band differential BPFs and Section 5 reviews wide-band and ultra-wideband differential BPFs, with tables of comparisons between these designs follow each section. Section 6 shows the challenges and future development of differential-fed microstrip BPFs. Finally, Section 7 presents the conclusion of our review.

## 2. Single-Band Differential Microstrip BPFs

**Figure 1:**
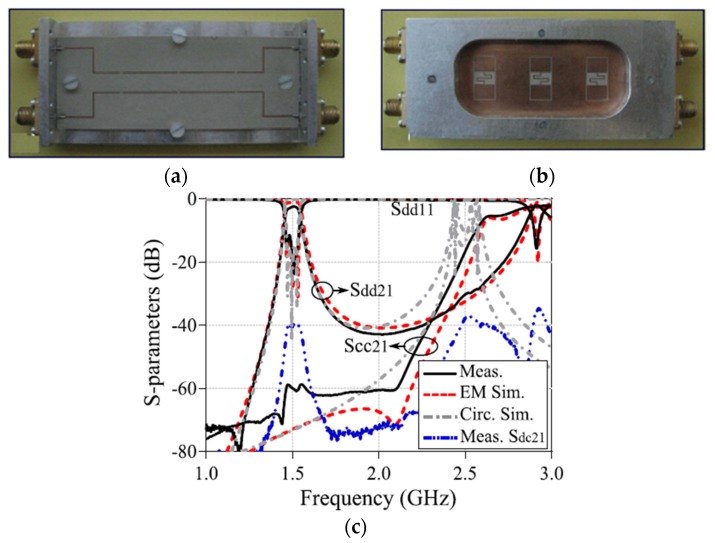
The proposed differential planar filter [42]: (**a**) Front view of the prototype; (**b**) Back view of the prototype; (**c**) Simulations and measurement results.

Recently, many single-band differential planar BPFs based on different techniques have been reported [39,40,41,42,43,44,45,46,47,48,49,50,51,52,53,54,55,56]. The main difference between these designs is the structures utilized. Several types of resonators and techniques can be used to obtain a single-band differential planar BPF with different performance. Generally, the common-mode noise suppression is an interesting topic for high-speed and high-frequency wireless applications. The common-mode noise signals can degrade the differential-mode transmitted signals as well as the entire power of such wireless applications. To suppress the common-mode signals, some researchers and engineers have proposed the use of a series of combinations of single-band differential-fed planar filters and transmission lines [39]. However, this technique will lead to a large area and so not suitable for new demands of compact systems. However, Ebrahimi et al. have proposed a new balanced BPF using dumbbell-shaped defected ground structures (DGSs) [42]. The proposed DGS resonator provides the option of implementing higher-order differential filters. Also, in comparison with similar techniques such as S-shaped complementary split-ring resonators (CSRRs), which have similar structures in common-mode and differential mode transmission [39], differential filters based on DGSs provide high common-mode attenuation by utilizing two separate equivalent models in differential- and common-mode operations. Figure 1 shows a photograph of the fabricated prototype with simulated and measured differential- and common-mode performance.

Furthermore, balun structures have an essential role in RF, MW and mmWave wireless applications to feed different differential components such as filters, antennas and power amplifiers (PA) by creating the differential mode signal [43]. According to this, a new technique is proposed to design a differential-fed planar BPF and balun filter by incorporating edge- and connected-couplings [43]. In this technique, the edge- and connected-couplings are utilized simultaneously to design differential-fed planar BPF and Balun filter. This combination provides lower insertion loss, higher common-mode rejection ratio (CMRR) and better stopband attenuation compared with some other design techniques presented previously.

**Figure 2:**
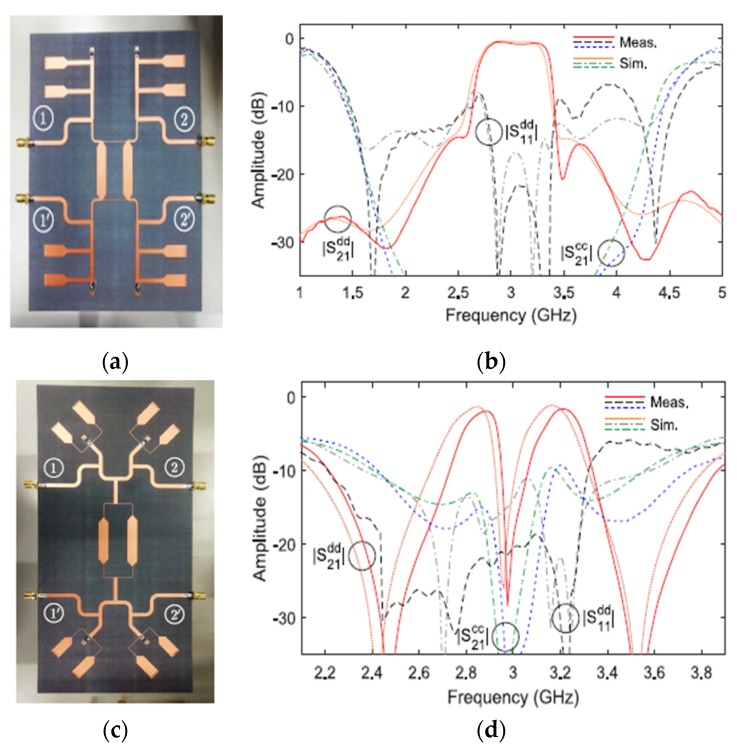
The proposed differential planar filters [44]: (**a**) Single-band filter configuration; (**b**) Single-band simulation and measurement results; (**c**) Dual-band filter configuration; (**d**) Dual-band simulation and measurement results.

Gómez-García et al. [44] presneted a new differential-fed planar filter based on asymmetrical quasi-reflectionless design technique. The filter uses quasi-absorptive resistively ended bandstop filter paths connected to the input and output terminals of the structure. With this case, the nontransmitted differential input power of the stopband signal is passed to the resistor loads of the bandstop terminals. This technique shows that the higher-order BPFs will lead to sharp differential-mode roll-off skirts rejection and higher suppression to the common-mode signals with the same quasi-absorptive mechanism. Figure 2 below shows fabricated prototypes of the differential-fed planar filters for single-band and dual-band configurations and their simulated and measured differential-mode performance.

**Figure 3:**
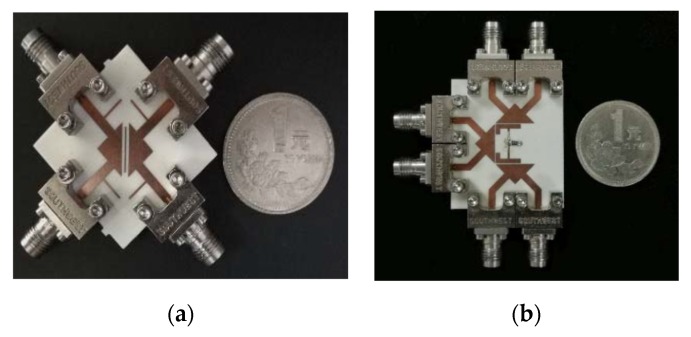
The proposed differential planar structure [45]: (**a**) Balanced BPF; (**b**) Balanced-to-balanced filtering power divider.

An isosceles right-angled triangular microstrip technique was proposed and developed recently by Rong et al. [45]. In this approach, a differential-fed bandpass filter, a balanced–to-balanced filtering power divider and a balanced–to-unbalanced (balun) bandpass filter resonating at 10 GHz were introduced. As shown in Figure 3a, the differential-fed bandpass filter consists of two triangular microstrip resonators and a half-wavelength (λ/2) resonator. To perform a novel design technique, the basic design in Figure 3a was modified to the new structure shown in Figure 3b to obtain the balance-to-balanced filtering power divider which is introduced by employing four resonators. The isolation between terminals 2 and 3 was improved by adding a λ/2 transmission line with a resistor between the main two λ/2 resonators.

**Figure 4:**
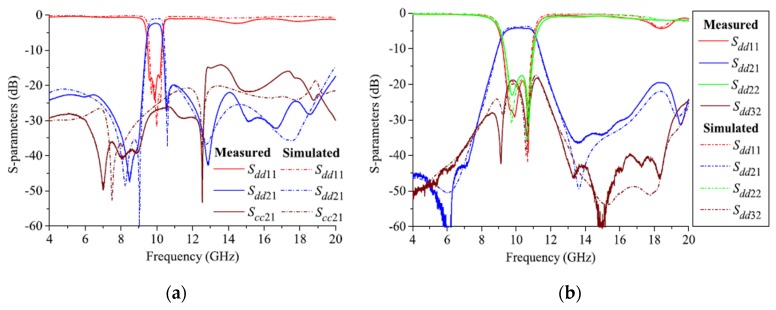
Simulated and measured s-parameters of the proposed design [45]: (**a**) Balanced BPF performance; (**b**) Balanced-to-balanced filtering power divider performance.

**Figure 5:**
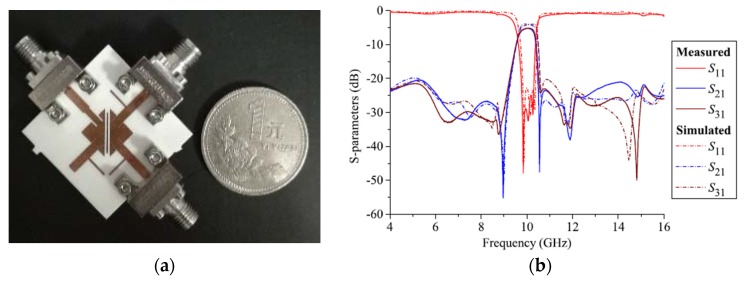
The proposed microstrip balun BPF [45]: (**a**) Photograph of the prototype; (**b**) S-parameter performance.

The simulated and measured s-parameters of the proposed balanced filter and balanced-to-balanced filtering power divider are shown in Figure 4a,b, respectively. Moreover, in this new study, converting the four ports differential filter to three ports circuit with one of the ports open is also introduced and applied to design a balun filter. A photograph of the fabricated balun bandpass filter using this technique is illustrated in Figure 5. Figure 5b shows the performance of the simulated and measured s-parameters of the proposed microstrip balun bandpass filter.

**Figure 6:**
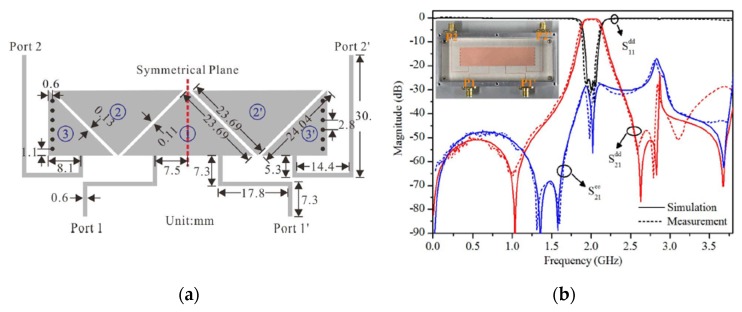
The proposed microstrip balun BPF [46]: (**a**) Photograph of the prototype; (**b**) S-parameter performance.

On top of that, further studies for this technique have been carried out by introducing a new approach [46]. The study was originating from a careful investigation on resonant characteristics of a right-angled isosceles triangular patch resonator, where a half-mode microstrip resonator with one electric wall and two magnetic walls in its three edges was established. Furthermore, a third-order differential fed bandpass filter with three finite transmission zeros was designed by accurately setting up the coupling coefficient factors between three right-angled isosceles triangular patch resonators and two half-mode right-angled isosceles triangular patch resonators [46]. Figure 6 shows the layout of the proposed third-order differential BPF and the hardware realization of the fabricated prototype with simulated and measured s-parameter responses.

Additionally, several papers have been published recently on investigating and implementing balanced microstrip filters based on substrate integrated waveguide (SIW) technology [47,48,49,50]. A compact balanced BPFs can be obtained by utilizing the dual-mode cavity resonators, which reduces the number of the resonators (filter’s order) by half [47]. Despite very few works that have been done in the literature using this technique, Hong-wei et al. have proposed a TM dual-mode cavity resonator for differential-fed BPFs. This technique can result in a compact structure with a sharp roll-off skirt rejection and an enhanced common-mode suppression over a wide frequency range.

**Figure 7:**
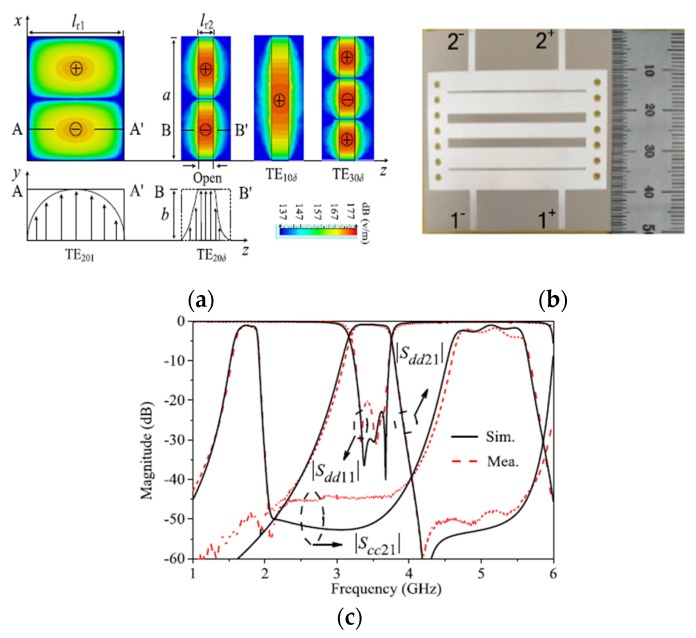
The proposed balanced BPF [50]: (**a**) Current distribution; (**b**) Photograph of the fabricated prototypes; (**c**) Simulated and measured s-parameters.

To obtain cascaded short-path TE20δ-mode resonators, a differential-fed SIW BPF loaded with transverse slots on the top layer of the structure is presented by Shen et al. [50]. This topology leads to a compact size planar BPF with low loss. Also, the presented balanced SIW filter shows a relatively wide 3-dB fractional bandwidth due to the introduced high coupling mechanism. Figure 7 shows the electric field distributions of the SIW resonators, a photograph of the fabricated balanced filter, and the simulated and measured s-parameter results.

**Figure 8:**
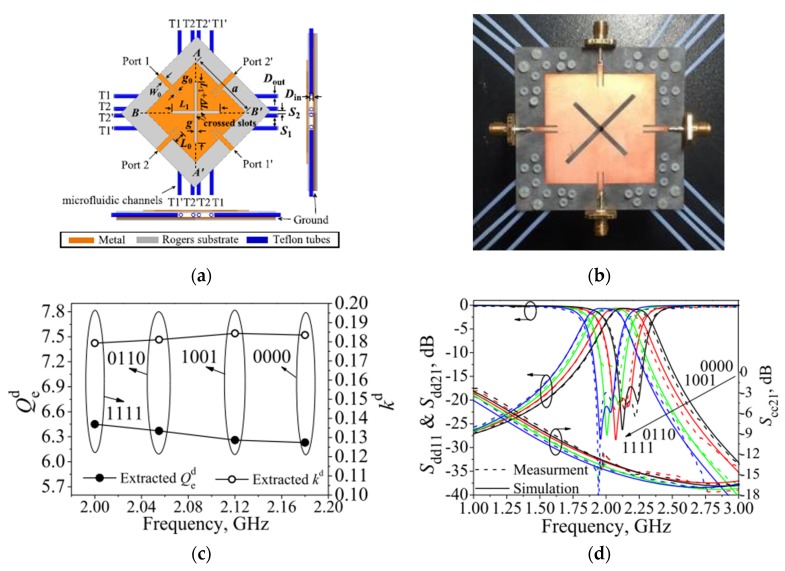
The proposed reconfigurable balanced BPF [35]: (**a**) Structure layout; (**b**) Prototype photograph; (**c**) External quality factors and coupling coefficients; (**d**) Simulated and measured tunable s-parameters.

High immunity to noise and crosstalk signals, which can be offered by the differential-fed structures, makes balanced components are widely used in many wireless communications. Therefore, and to achieve the requirements of high-performance tunable/reconfigurable RF devices, it is highly recommended to develop and investigate the design of tunable BPFs with differential-fed ports to improve the integration properties [35,51,52,53]. During the past few years, fluidics-based resonating technologies have been widely applied in tunable filter circuits [35,54,55,56]. Zhou et al. [35] presented a novel microfluidics-based reconfigurable differential-fed microstrip filter with accurately tuned passband transmission. The design is a second-order balanced structure and is mainly utilizing dual-mode transmission line resonators, as shown in Figure 8. The microfluidic circuit was introduced by employing a Teflon tube placed between the top and ground layers. The differential mode passband with a constant fractional bandwidth was tuned by filing water into the Teflon tube, and the low loss characteristics of the differential mode transmission band were achieved at each configuration. The enhanced characteristics could make the presented microfluidic balanced BPF a very promised application for the current and future wireless communications. Figure 8c illustrates the extracted external quality factor and coupling coefficients for all configuration states of the tunable balanced BPF, while Figure 8d shows the measured and simulated tunable s-parameter performance. The small frequency discrepancies between measurement and simulations are due to unpredictable fabrication tolerance as can be explained by Outerelo et al. [57]. Table 1 summarises performance comparisons for the presented techniques of single-band differential microstrip BPFs.

## 3. Dual-Band Differential Microstrip BPFs

Unlike single-band differential microstrip BPFs, fewer numbers of balanced filter designs and techniques have been proposed recently with a dual-band performance [58,59,60,61,62,63,64,65,66,67,68,69,70]. Nevertheless, increasing demand for flexible resonant devices has led to more attention and interest in designing and implementing of dual-/tri-/quad-band BPFs. Furthermore, more multi-functional devices and highly efficient design techniques are required to achieve better differential-mode characteristics and higher common-mode rejection ratio. However, and to obtain high selectivity, high-order dual-band balanced microstrip BPFs can be used. Unfortunately, these filters present high insertion loss due to increasing the inherent losses (metal resistance) [71]. In this section, we survey the main important and recent dual-band balanced planar BPF design techniques and its challenges.

**Figure 9:**
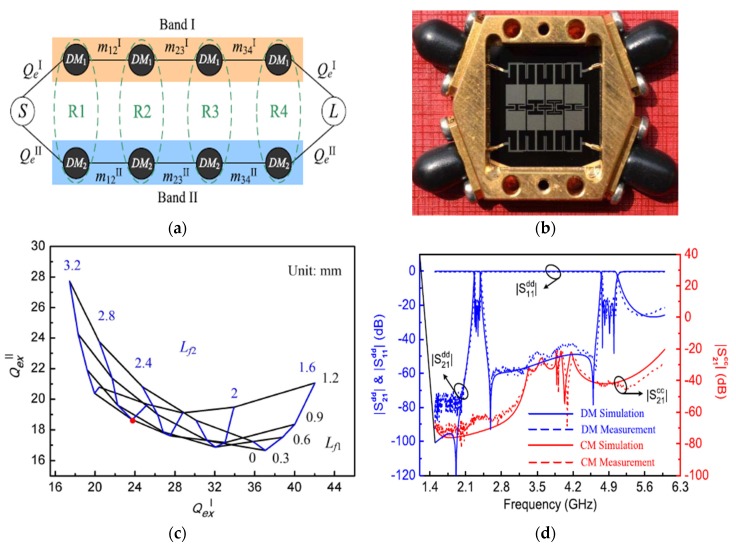
The proposed balanced four-pole dual-band BPF [62]: (**a**) Coupling scheme; (**b**) Photograph of the fabricated prototype; (**c**) Simulated external quality factors; (**d**) Simulated and measured frequency responses.

To overcome this challenge, balanced dual-band BPF based on high-temperature superconducting material technology was first proposed by the research group in [61,62,63]. Using this technology can offer highly efficient performance which is not affordable by using traditional materials. Ren et al. [62] proposed a balanced dual-band microstrip BPF based on the superconducting technology and using multi-mode close-loop stepped-impedance resonators. As shown in Figure 9a,b, the filter was firstly designed by using a conventional square ring loaded resonator and investigated with the transmission line model. Then, the stepped-impedance resonator structure was utilized to increase the design degree of freedom by controlling the differential mode performance. Finally, fourth-degree balanced BPF was introduced by modifying the previous structure and by applying a high-temperature superconducting technique. Figure 9b,c shows the performance of the proposed dual-band superconducting BPF. It is shown that controlling the feeding points L_f1_ and L_f2_ for ports 1 and 2 can improve the transfer of the maximum power between input and output ports. Also, it is shown that the presented technique provides good common-mode suppression of more than 20 dB in the transmission passband.

**Figure 10:**
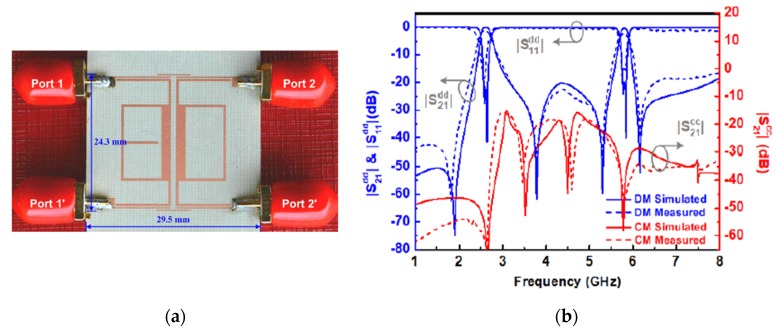
The proposed balanced dual-band BPF [66]: (**a**) Coupling scheme; (**b**) Photograph of the fabricated prototype; (**c**) Simulated external quality factors; (**d**) Simulated and measured frequency responses.

Ren et al. [66] presented a compact dual-band balanced microstrip BPF based on quadruple-mode stepped-impedance closed-loop resonators by the same research group. Two differentially excited modes of stepped-impedance close-loop resonators were employed to produce the dual differentially-def bands. The admittance ratio factor of the stepped-impedance resonators was adjusted accurately to stop the interference of the common-mode signal with the differential-mode one. To improve the common-mode attenuation within the differential transmitted signal and to enhance the roll-off skirts of the differential-mode signal, two compact and adjacent transmission lines were loaded to the input and output feeding ports as seen in Figure 10a. The source-load coupling scheme was introduced and studied to improve the selectivity and to generate more finite transmission zeros. As seen in Figure 10b, four transmission zeros successfully generated and this has improved the stop-band selectivity for the differential mode performance. Furthermore, the predicted minimum common-mode insertion losses for the first and second bands were 62 dB and 48, respectively. Moreover, it can be noticed that the presented balanced dual-band filter has better performance than the proposed designs by Wei et al. [64] and Chen et al. [65] in regards to the common-mode suppression and the stopband rejection of the differential mode performance.

**Figure 11:**
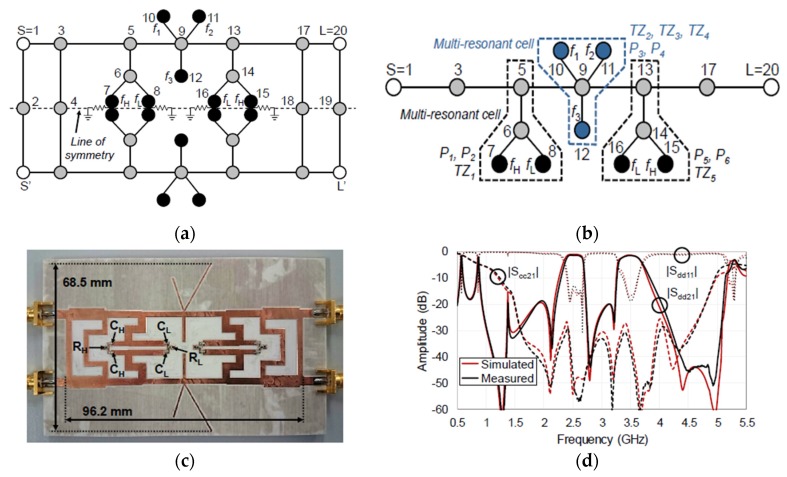
The proposed balanced dual-band BPF [65]: (**a**) Coupling scheme of the proposed filter; (**b**) Differential-mode single-ended coupling scheme; (**c**) Photograph of the fabricated prototype; (**d**) Simulated and measured frequency responses.

A new elliptic-type balanced dual-band BPF was presented by Simpson et al. [69]. To improve the filter performance, coupling scheme synthesis was introduced and discussed in this paper as shown in Figure 11a. In this technique, multi-resonant circuits were connected in series for elliptic-type structure in the differential-mode excitation to enhance the common-mode rejection ratio and improve the stopband selectivity. It should be noted that the coupling-route diagram in Figure 11a can be utilized only for tackling the actual number of resonators and coupling circuits in the balanced bandpass filter and can not be achieved form the single-ended coupling-route diagram presented in Figure 11b. Figure 11c,d show a photograph of the hardware realization and the frequency responses of the proposed balanced dual-band BPF, respectively. Compared with other similar structures [67,68], the presented techniques can result in higher selectivity and better stopband rejection.

Karimi et al. [72] proposed a new coupling system named unequal two coupled U-shaped structure (TCUS) and applied to design a dual-band differential microstrip BPF with independently controllable passbands. To improve the suppression level in the differential mode and the common-mode rejection ratio, third-order Sierpinski fractal design was utilized on the I-shaped transmission line. As a result, the proposed filter has a differential mode return loss better than 15 dB in the and a common-mode suppression level higher than 16 dB. Also, the filter has a high rejection level of 30 dB in the upper stopbands with insertion losses better than 0.6 and 1.8 dB in the first and second bands, respectively. Table 2 summarises performance comparisons for the presented dual-band differential microstrip BPFs.

## 4. Tri- and Quad-Band Differential Microstrip BPFs

Although the responses of the balanced microstrip filters presented in the previous two sections are quite sufficient, they are only useful for single-band and dual-band applications. Therefore, to meet the increasing demands for multi-band wireless systems, several tri-band and quad-band balanced microstrip filters have been proposed in the past few years [65,66,73,74,75,76,77,78,79]. In this section, we survey the main design techniques recently proposed for tri-band and quad-band balanced microstrip BPFs.

**Figure 12:**
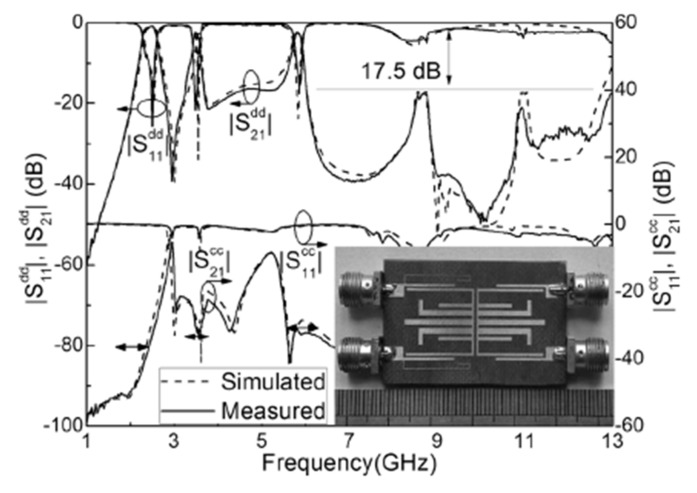
Simulated and measured frequency responses of the balanced tri-band BPF [64].

**Figure 13:**
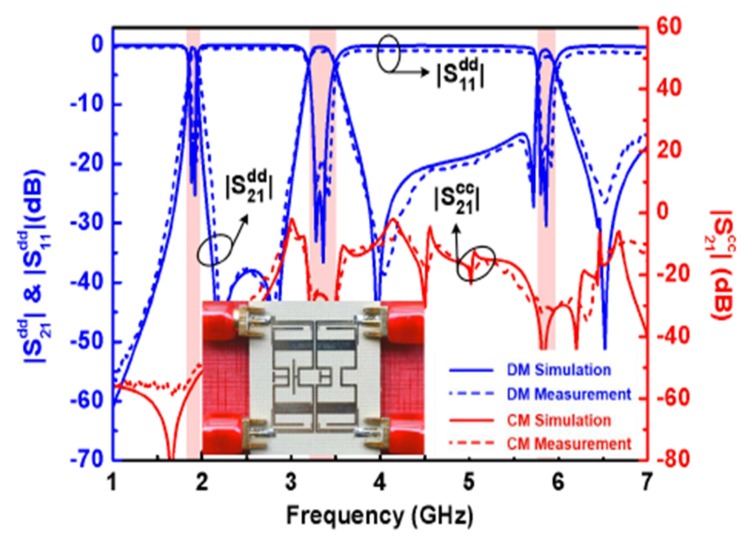
Simulated and measured frequency responses of the balanced tri-band BPF [66].

The same dual-band microstrip balanced filter structures presented by Wei et al. [64] and Ren et al. [66] have also been modified and developed to resonate at three differential-mode passbands. A differential-fed BPF has been implemented based on five stub loaded resonators, as shown in Figure 12 [64]. Also, two parallel λ/2 open stub transmission lines were used at the input and output ports of the modified balanced structure, which can generate one extra transmission zero between the dual-band passbands. Thus, the roll-off skirt sharpness has been improved, as seen in Figure 12. The stopband bandwidth was enhanced to 13 GHz with a suppression level of more than 17 dB, which is five times the first-mode operation frequency. A differential-fed BPF has been designed based on two coupled six-mode stepped-impedance close ring loaded transmission lines, as shown in Figure 13 [66]. To obtain a compact size structure, the four open-loop and center transmission lines of the stepped-impedance ring resonators have been folded. In a similar way, and to shift the common-mode frequencies without affecting the differential-mode performance, three T-shaped transmission lines were employed at the center point of both stepped-impedance ring resonators. A photograph of the fabricated prototype with the simulated and measured frequency responses is presented in Figure 13.

**Figure 14:**
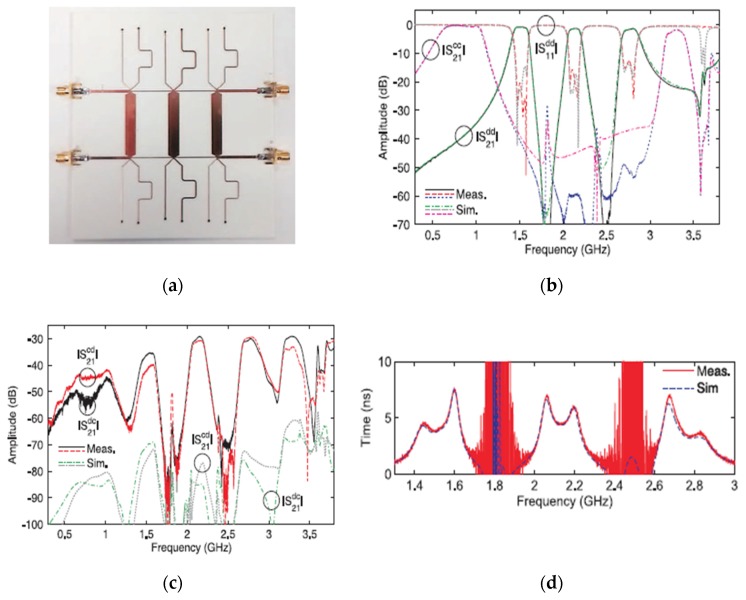
The proposed balanced tri-band BPF [73]: (**a**) Photograph of the fabricated prototype; (**b**) Differential-mode performance; (**c**) Common-mode performance; (**d**) Group delay.

A multi-stub-loaded quasi-elliptic-type technique has been used in the design of new class tri-band balanced microstrip BPF [73]. A detailed structure of the introduced filter is shown in Figure 14a. To realize a differential-mode filtering response, each side of the symmetry line has three cascaded circuits of six-stub-loaded cells with two transmission lines. Additionally, to increase the common-mode rejection ratio for the three differential passbands, two of the stub lines of each circuit were short-circuited to the ground layer, while the other stub lines were connected to the virtual ground utilized by the differential-fed ports. The simulated and measured frequency responses for the differential-mode, common-mode and group delay are given in Figure 14b–d, respectively. Compared to the filter designed by Wei et al. [64], this filter has higher common-mode rejection levels and fewer insertion losses for the three passbands with higher bandwidths for the second and third passbands. Furthermore, the presented technique has another attractive performance which is the lack of electromagnetic couplings between the transmission lines, and this has led to the low insertion loss, simple design, and possibility for lumped-element transformation.

**Figure 15:**
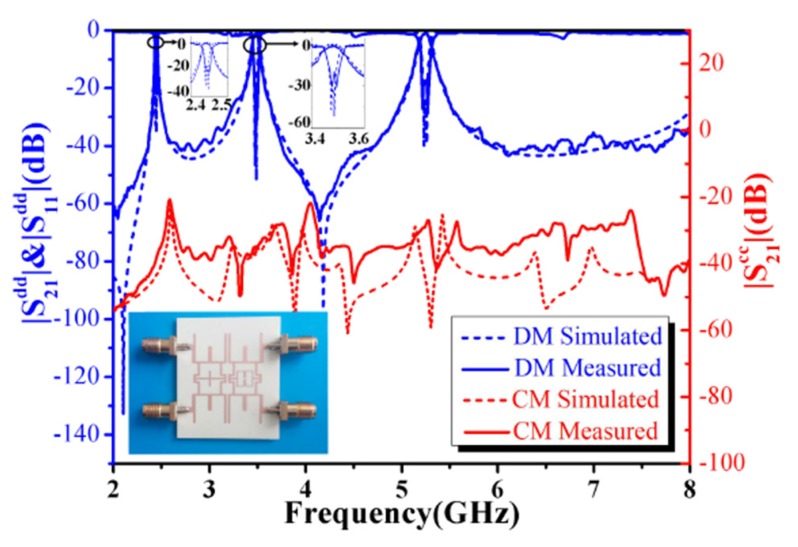
Simulated and measured differential- and common-mode performance of the balanced tri-band BPF with a photograph of the fabricated prototype [74].

The square ring loaded resonator is a simple technique and suitable for multi-band wireless applications. The design of tri-band differential BPF with a high common-mode rejection level and wide-upper stopband bandwidth using a square ring resonator technique was recently presented in [74]. In this work, the balanced BPF involves a ring resonator and six-loaded-stub lines, and its frequency response was achieved by even- and odd-mode analysis. Under differential-mode excitation, the multi-band performance of the presented square ring resonator was investigated and utilized to construct tri-differential bands with a wide stopband rejection bandwidth. After this step, the design was loaded with stub lines along with the symmetry line to enhance the common-mode rejection ratio. A photograph of the fabricated prototype with the simulated and measured differential- and common-mode s-parameters of the proposed tri-band balanced BPF is shown in Figure 15. Also, the filter has shown a reasonable degree of freedom to control the differential-mode passband. According to the achieved performance, and due to the attractive tri-band differential-mode frequency response and wideband common-mode rejection characteristics achieved by this technique, the presented differential-fed BPF has an excellent perspective on multi-mode wireless devices.

**Figure 16:**
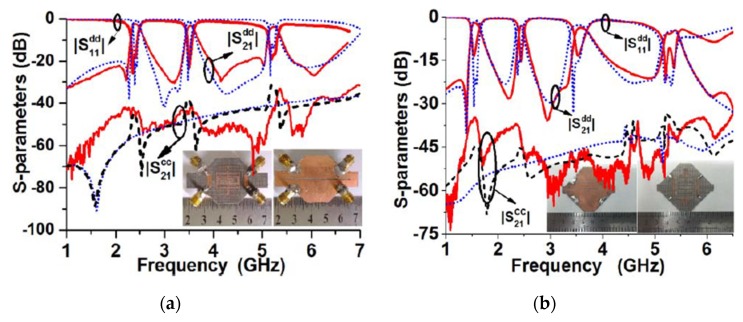
Simulated and measured results and photographs of the prototypes [77]: (**a**) Tri-band differential-fed BPF; (**b**) Quad-band differential-fed BPF.

Besides, complementary split-ring resonators [75] and octo-section stepped-impedance ring resonator [76] have also been introduced in the literature as new topologies for the designing of tri-band balanced BPF. Zhang et al. [77] introduced a novel differential-fed tri- and quad-band microstrip BPFs with controllable bandwidths using a slotline coupling feed technique. Figure 16 shows the simulated and measured differential- and common- mode frequency responses and a photograph of the hardware realization of the proposed tri- and quad-band balanced BPFs. For differential-mode excitation, the tri- and quad-band responses were obtained by three- and four-λ/2 resonators, respectively, which are utilized for specified resonant frequencies. In addition, the coupling coefficient factors and external quality factors of each differential-mode resonant frequency have been adjusted independently and thus, the operational bandwidth of each bandpass has been also controlled. It is worth mentioning that the presented quad-band differential-fed BPF was the first-ever introduced in the literature.

**Figure 17:**
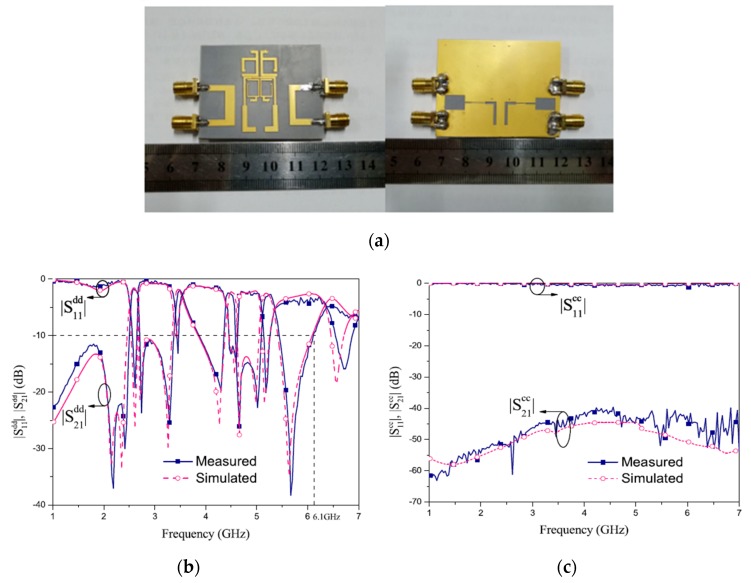
The proposed balanced quad-band BPF [78]: (**a**) Photograph of the fabricated prototype; (**b**) Differential-mode performance; (**c**) Common-mode performance.

Also, a differential-fed quad-band microstrip BPF with adjustable center frequencies and bandwidths using slotline technique was presented by Wei et al. [78]. In the differential-mode excitation, the resonant frequencies of the four passbands have been adjusted by altering the electrical length ratio of each asymmetric short stub-loaded resonator while the operational bandwidths have been controlled by changing the gap dimensions between the resonators itself and the interdigital transmission lines. Figure 17 shows a photograph of the prototypes and simulated and measured performance of the proposed quad-band balanced filter. Table 3 summarises performance comparisons for the presented papers in the literature with the scope of tri- and quad-band differential microstrip BPFs.

## 5. Wide-Band and Ultra-Wideband Differential Microstrip BPFs

The research on wide-band and ultra-wideband systems is an attractive topic for current and future wireless applications due to the preferable functions to deal with high data rate transmissions. Wide-band and ultra-wideband BPFs are one of the fundamental elements of wide-band MW/RF communications, which has been deeply investigated in the literature [79,80,81,82,83,84,85,86]. Nevertheless, there has been little attention paid to wide-band and ultra-wideband differential microstrip BPF designs in the past few years [87,88,89,90,91,92,93,94,95,96]. Despite the good common-mode attenuation that has been obtained in these filters, the roll-off skirts and insertion losses of the differential mode passbands still require some improvements.

**Figure 18:**
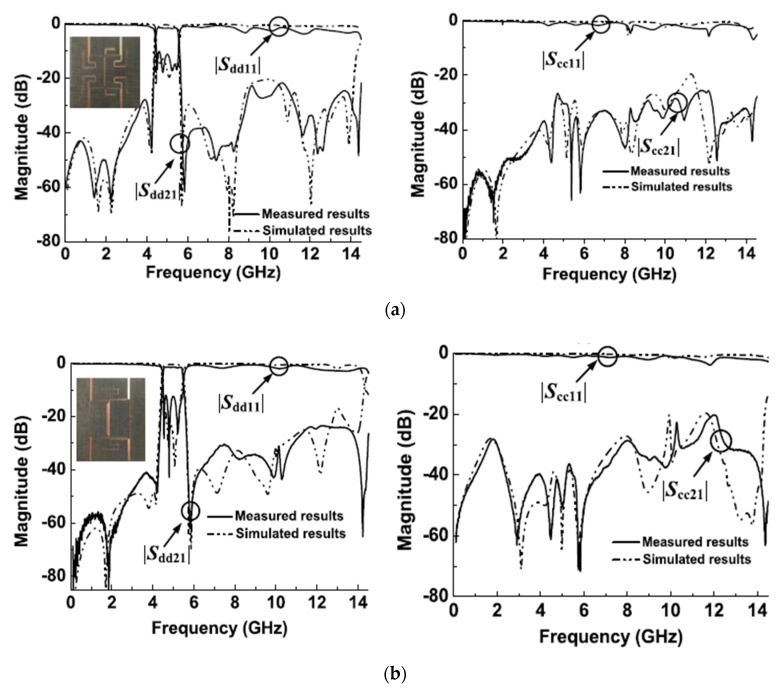
Photographs for the prototypes and s-parameter results of proposed balanced wide-band BPFs [87]: (**a**) Firat design; (**b**) Second design.

Recently, some design techniques for balanced wide-band microstrip BPFs have been introduced [87,88,89,90,91]. One technique is using input-/output-coupled lines with open- and shorted-circuit transmission stubs [87], which can provide sharp roll-off rejection and high stopband suppression levels. According to this, two new wide-band differential-fed microstrip BPFs have been proposed. The first wide-band differential filter utilized by four shunt-connected λ/2 transmission lines as shown in Figure 18a. Even and odd mode analysis was carried out and four finite transmission zeros were successfully generated for the differential- and common-mode excitation. The second wide-band differential filter based on asymmetric open- and short-circuit stubs by replacing the two λ/4 transmission lines of the first filter by two λ/2 resonators, as shown in Figure 18b. For the common-mode operation, five finite transmission zeros have been realized and thus the stopband rejection level has been enhanced. Figure 18a,b shows the photograph of the fabricated prototypes and the s-parameter performance for the presented wide-band balanced BPFs. A similar approach has been applied to design a simple structure wide-band balanced BPF using three λ/2 transmission lies resonators [88]. The new technique does not require two symmetric circuits along the central line of the structure and therefore a compact size has been obtained.

**Figure 19:**
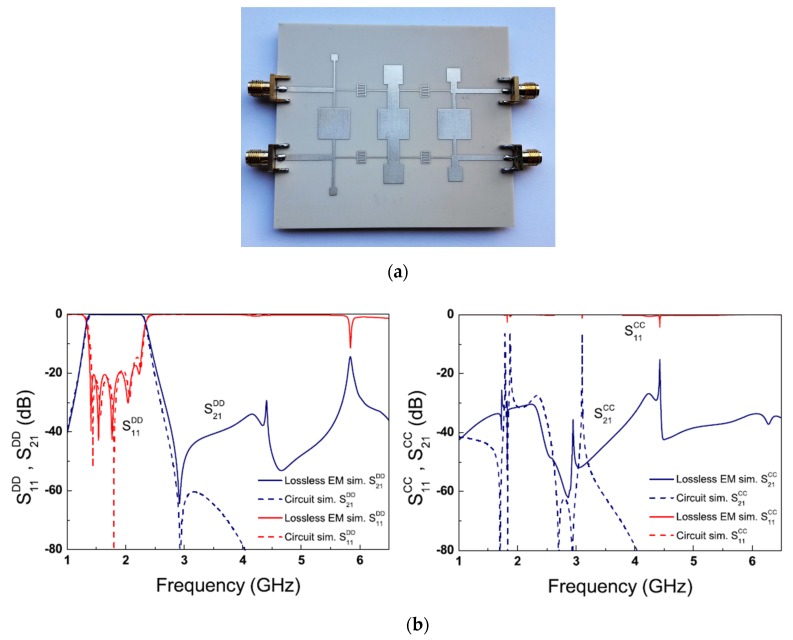
The proposed wide-band balanced BPF [89]: (**a**) Photograph for the prototype; (**b**) S-parameter performances.

Sans et al. [89] proposed a compact wide-band differential-fed BPF with wide-stopband restriction for the common- and differential-modes and based on integrating multisection stepped-impedance resonators with interdigital capacitors. This technique combines an aggressive-mapping optimization (that transforms the components of the electronic circuit into the required filter structure) with pre- and post-optimization algorithms required to find the best position of the finite transmission zeros. Filter size and the common- and differential-mode wide-band rejection characteristics are the main advantages of the presented technique. Figure 19a shows a photograph of the prototype differential-fed BPF which was obtained by using photomask etching. Figure 19b illustrates the simulated and measured s-parameters for the differential- and common-mode operation of the presented wide-band BPF.

**Figure 20:**
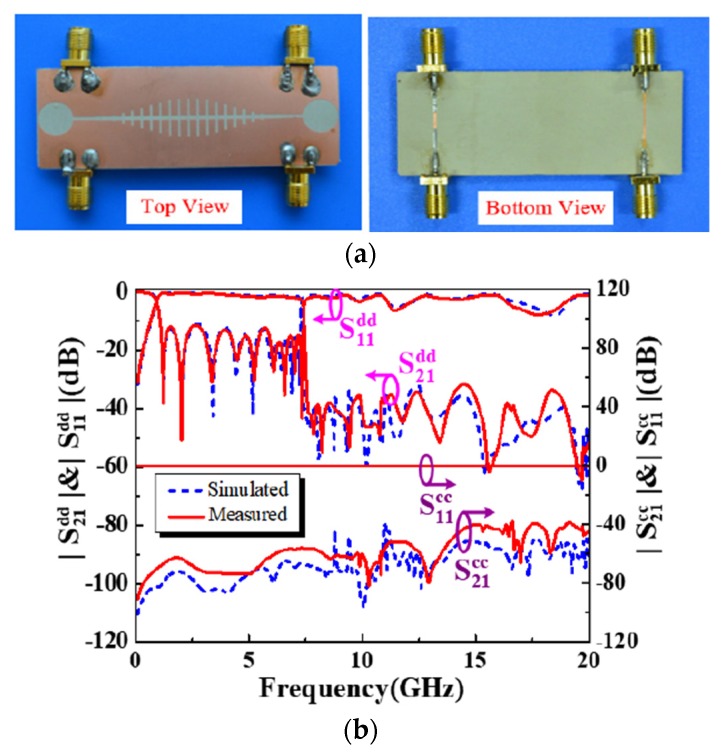
The proposed wide-band balanced BPF [91]: (**a**) Photographs for the prototypes; (**b**) S-parameter performances.

More recently, a compact wide-band balanced BPF based on coupled line resonators and two pairs of lumped capacitors has been presented by Dong et al. [90]. The loaded capacitors have been combined with the even- and odd-mode impedances of the coupled transmission lines and this has led to the generation and adjustment of three transmission poles and four finite transmission zeros under the differential-mode excitation and three finite transmission zeros under the common-mode excitation. On top of that, and unlike the previously reported technique, the surface plasmon polaritons technique is also another new technique that has been reported by Liu et al. [91] for wide-band balanced BPFs. The surface plasmon waveguide has lowpass and slow-wave properties, while the microstrip patch has a highpass response with intrinsic common-mode suppression properties. Integrating these two components will lead to a new wide-band balanced BPF with improved common-mode rejection ratio and better stopband differential-mode performance as proved in this paper. The presented filter is symmetric along the centerline of the design with differential-fed ports. Longitudinal slots were loaded on the ground layer, and this provides a mode conversion from microstrip patch to surface plasmon polaritons waveguide. Figure 20 shows a photograph of the fabricated prototypes and simulated and measured s-parameters for the proposed balanced wide-band BPF.

**Figure 21:**
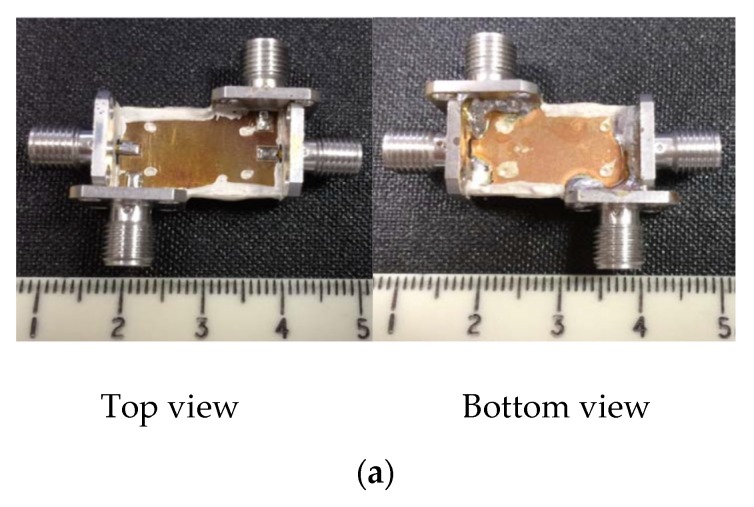

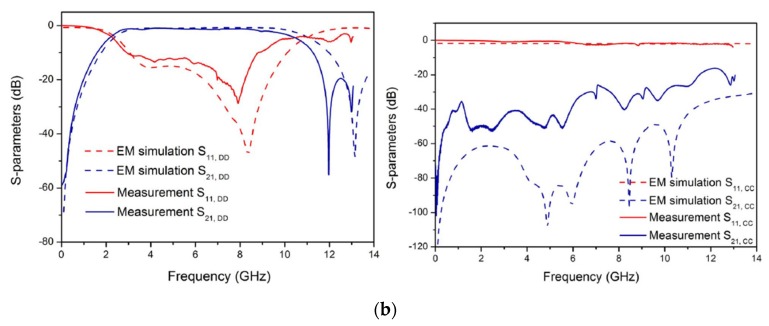
The proposed wide-band balanced BPF [93]: (**a**) Photographs for the prototypes; (**b**) S-parameter performances.

On the other hand, few numbers of design techniques for balanced ultra-wideband microstrip BPFs have been reported recently [92,93,94,95,96]. One technique was presented by using half mode dumbbell defected ground structure to design a compact ultra-wideband differential-fed BPF [92]. In this paper, a T-shaped multimode resonator with a short-circuit stub was used to obtain the ultra-wideband performance. Also, a half-mode defected ground structure was presented to the patch layer to obtain a compact design with a wideband common-mode rejection performance. Moreover, another ultra-wideband differential-fed BPF based on a low-cost liquid-crystal polymer material was recently presented by Aliqab et al. [93]. The main target of this technique is to design new, cheap, compact and simple balanced BPF by cascading two baluns structures. Figure 21 illustrates a photograph of the fabricated prototypes and simulated and measured s-parameters for the proposed balanced ultra-wideband BPF. Table 4 summarises performance comparisons for the recently proposed wide-band and ultra-wideband differential microstrip BPFs.

## 6. Challenges of Balanced Microstrip BPFs and Future Development

Over the last few years, RF designers, researchers, and engineers have investigated balanced/differential microstrip BPF design techniques as alternatives to the existing approaches and topologies to develop high differential- and common-mode performances. Compared to the single-ended BPFs, some essential challenges are accompanying double-ended (balanced) BPFs, which have both differential-mode excitation of improved stopband suppression and common-mode excitation of enhanced common-mode rejection ratio. As can be observed from the previous sections of this paper, if the bandwidth of the differential-mode response increased, the common-mode rejection ratio will be decreased, and this can be considered as a common challenge for all differential-fed BPFs. To overcome this challenge, some differential-fed BPFs with a wideband common-mode rejection ratio by employing dual-mode ring resonators were introduced in [35,37,48,49]. Also, since the differential-fed filters should be designed with symmetrical structure, therefore these filters should be two times the size of the single-ended ones, and thus the size reduction will be an essential challenge for the balanced BPFs.

For multi-band balanced BPFs, several techniques have been used such as stepped-impedance, coupled-line and substrate integrated waveguide resonators. However, the filter presented by Liu et al. [76] using an octo-section stepped-impedance resonator can offer the advantage of a high common-mode rejection ratio with wide-stopband suppression. Nevertheless, the isolation between the adjacent bands as well as the roll-off rejection should be further improved to meet the prospective high-performance specifications of the current and future wireless applications. Also, classical high-order multi-band differential-fed filters present high insertion loss performance because of the inherent copper resistance. This problem has also been solved by using high-temperature superconducting techniques to obtain a very promised performance which is not affordable using traditional materials [63,64].

Wide-band and ultra-wideband differential microstrip BPFs are essential and essential components of the future wide-band wireless applications to tackle the high speed and high data rate transmissions. For these BPFs, it is noticed that the size, insertion losses, and differential-mode bandwidth should also be taken into consideration and carefully investigated by the designers. Most of the proposed wide-band and ultra-wideband balanced BPFs are designed based on single-layer microstrips. Therefore, it should be pointed out that using liquid crystal resonators and low-temperature co-fired ceramic can enhance the common- and differential-mode suppression, thus improving and developing the performance of the wide-band communication systems [93,94,95,96,97].

Differential-fed BPFs based on substrate integrated waveguide techniques can be used for mmWave applications to obtain lower losses and higher quality factors and more power handling capability when compared with traditional planar BPFs [47,48,49,50]. Nevertheless, using these techniques can present some challenges, such as the improvement of bandwidths and reduction of the losses and sizes of the filters. From the presented review, the design technique presented by Shen et al. [47] can overcome these challenges by using only one single-layer half-mode substrate integrated waveguide resonator with four slots. As one of the microwave components, differential-fed microstrip BPFs can also be designed, analyzed, and optimized using artificial intelligence, neural networks, and bio-inspired optimization techniques [98,99]. These approaches can be utilized for future differential-fed microstrip BPF designs since these double-ended structures require more analysis and parameter studies than single-ended structures. Therefore, using these approaches can lead to overcoming many of these challenges by considering and dealing with many variables simultaneously. It is anticipated that more novel fully balanced microstrip BPFs will be seen in the near future.

## 7. Conclusions

Up-to-date detailed reviews of differential-fed (balanced) microstrip BPF design techniques, challenges, and future developments are presented in this paper. Single-,dual-,tri-, and wide-band differential-fed microstrip BPFs are surveyed, which employ several design techniques for current and future wireless applications. A comparison between different design techniques and structures is also presented and discussed in this paper by focusing on the main important and recent contributions in the balanced microstrip BPFs. Compared with the single-ended BPFs, the presented balanced designs have the advantages of high common-mode wideband attenuation, high noise immunity, high passband selectivity, and wide-stopband harmonic suppression with low levels of radiation power loss in wireless systems. From these reviews, we have also concluded the main challenges and future developments of balanced microstrip BPFs filters. Despite certain limitations, we anticipate that more new, promising, and multifunctional differential-fed BPFs will be seen in the next few years.

## Figures and Tables

**Table 1:** Performance comparisons for some recent single-band differential microstrip BPFs.

Ref.	Technique	Freq. (GHz)	FBW (%)	IL (dB)	CMS (dB)	DMSS (dB)	Size (λ_g_ × λ_g_)
**[35]**	Microfluidcally tunable	2.1	21.8	0.6	>18	---	0.32 × 0.32
**[42]**	Dumbbell	1.5	6	2.4	>30	>30 @ ≤1.55 *f*_0_	0.34 × 0.34
**[44]**	Quasi-reflectionless	3	21	1.7	>30	---	1.69 × 1.15
**[45]-I**	Isosceles balanced	10	6	2.35	>26	>20 @ ≤2.0 *f*_0_	0.94 × 0.94
**[45]-II**	Isosceles FPD	10	18	4	>30	>20 @ ≤2.0 *f*_0_	0.94 × 0.94
**[45]-III**	Isosceles balun	10	6	5	---	>20 @ ≤1.6 *f*_0_	0.94 × 0.94
**[49]**	Right-angled isosceles	2	12.9	0.5	>27.8	>26 @ ≤2.0 *f*_0_	0.51 × 0.25
**[50]**	Substrate integrated waveguide	3.5	16	0.91	---	>20 @ ≤1.7 *f*_0_	1.2 × 0.83

IL: insertion loss; CMS: common-mode suppression; DMSS: different-mode stopband suppression; FBW: fractional bandwidth; FPD: filtering power divider.

**Table 2:** Performance comparisons for some recent dual-band differential microstrip BPFs.

Ref.	Technique	Freq. (GHz)	FBW (%)	IL (dB)	In-Band CMS (dB)	Number of TZs	Size (λ_g_ × λ_g_)
**[45]**	Quasi-reflectionless	2.85/3.15	5.2/5.1	1.9/1.7	20/25	3	2.0 × 0.96
**[58]**	Stub-loaded SIRs	2.45/5.25	9.8/4.6	2.4/4.6	53/45	3	0.38 × 0.42
**[59]**	Coupled SIRs	2.4/5	16.4/8.6	1.78/2.53	32/32	3	0.50 × 0.70
**[60]**	Substrate integrated waveguide	9.47/9.96	2.9/3.1	1.89/1.73	31/30	3	2.87 × 2.95
**[62]**	High temperature superconducting	2.32/4.90	3.9/4.9	0.13/0.16	63/40	5	0.32 × 0.31
**[64]**	Stub-loaded SIRs	2.5/5.8	12.9/4.5	0.77/1.56	42/38	4	0.15 × 0.37
**[65]**	Quasi-elliptic	2.5/3.5	13/12	1.3/1.4	52/38	5	0.80 × 0.57
**[66]**	Quadruple-Mode SIRs	2.6/5.8	10.2/3.6	1.1/2.15	62/48	4	0.26 × 0.34

IL: insertion loss; CMS: common-mode suppression; FBW: fractional bandwidth; TZs: transmission zeros; SIR: stepped-impedance resonator.

**Table 3:** Performance comparisons for some recent tri- and quad-band differential microstrip BPFs.

Ref.	Technique	Freq. (GHz)	FBW (%)	IL (dB)	In-Band CMS (dB)	Number of TZs	Size (λ_g_ × λ_g_)
**[64]**	Stub-loaded SIRs	2.5/3.5/5.8	13.2/3.1/3.5	0.8/2.3/2.4	32/31/32	6	0.18 × 0.38
**[66]**	Quadruple-Mode SIRs	1.9/3.3/5.8	4.74/8.6/2.78	0.94/1.2/1.93	54/27/32	5	0.91 × 0.23
**[73]**	Multi-stub-loaded	1.5/2.1/2.7	12/7.3/7	0.74/1.3/1.4	37/59/48	7	0.74 × 0.74
**[74]**	Multi-mode SRLR	2.4/3.5/5.2	1.2/2/1.52	0.71/0.9/0.67	38/32/25	6	0.56 × 0.43
**[77]-I**	Slotline coupled-feed	2.4/3.5/5.2	9/5.5/4	2.4/3.5/3.6	33/33/33	6	0.30 × 0.25
**[77]-II**	Slotline coupled-feed	1.54/2.15/3.6/5.2	11/4.9/8.8/5	1.9/2.8/3.7/4.6	>32	5	0.45 × 0.32
**[78]**	Balanced MS transition	2.54/3.46/4.5/5.2	6.45/5.68/3.93/4.91	1.58/1.78/2.23/2.45	>41	8	0.59 × 0.42

IL: insertion loss; CMS: common-mode suppression; FBW: fractional bandwidth; TZs: transmission zeros; SIR: stepped-impedance resonator; SRLR: square ring loaded resonator; MS: microstrip/slotline.

**Table 4:** Performance comparisons for some recent wide-band and ultra-wideband differential microstrip BPFs.

Ref.	Technique	Freq. (GHz)	FBW (%)	IL (dB)	CMS (dB)	Stopband (dB)	Size (λ_g_ × λ_g_)
**[87]-I**	Symmetrical/asymmetrical coupled lines	5	30	< 2	>4 5	>20 @ ≤2.9 *f*_0_	0.65 × 0.60
**[87]-II**	Symmetrical/asymmetrical coupled lines	5	25	< 2	>50	>25 @ ≤2.9 *f*_0_	0.65 × 0.50
**[88]**	Half-Wavelength Lines	1.8	57.8	< 0.27	>20	>20 @ ≤0.67 *f*_0_	0.26 × 0.26
**[89]**	Multisection mirrored SIRs	1.8	55.4	< 1	>28	>22 @ ≤3.6 *f*_0_	0.48 × 0.51
**[91]**	Slotline surface plasmon polaritons	4.2	147	< 1.7	>55	>30 @ ≤6 *f*_0_	2.95 × 0.89
**[93]**	Liquid-crystal polymer	6.85	118	< 1.1	>35	---	0.94 × 0.94

IL: insertion loss; CMS: common-mode suppression; FBW: fractional bandwidth; SIRs: stepped-impedance resonators.
